# Assessing attitude towards condom use among truck drivers at transhipment location, Meerut District, India, using multidimensional condom attitude scale

**DOI:** 10.4314/ahs.v22i4.35

**Published:** 2022-12

**Authors:** Ashish Pundhir, Arvind Shukla, Manoj Kumar Gupta, Akhil Dhanesh Goel, Pawan Parashar, Amit Mohan Varshney

**Affiliations:** 1 Department of Community Medicine and Family Medicine, All India Institute of Medical Sciences Kalyani Kalyani, West Bengal, India- 741245; 2 Department of Community Medicine and Family Medicine, All India Institute of Medical Sciences Raipur Great Eastern Road, Tatibandh Raipur, Chhatisgadh, India – 492099; 3 Department of Community Medicine and Family Medicine, All India Institute of Medical Sciences Jodhpur, Basini, Industrial Phase 2 Jodhpur, Rajasthan, India -342005; 4 Department of Community Medicine Subharti Medical College, Delhi Haridwar Bypass Road, Meerut, Uttar Pradesh – 250005, India; 5 Department of Community Medicine GMC, Sahranpur Uttar Pradesh – 247232 India

**Keywords:** Unsafe sex, condom use, India

## Abstract

**Background:**

In India, unsafe sex has been documented among truck drivers. This study explores the factors influencing their attitude of trucker towards condom use.

**Methods:**

A cross-sectional study design was adapted for this study in which 25 factors were chosen to assess attitude toward condom use on 7 point validated Likert scale UCLA Multidimensional Condom Attitude Scale on 5 subcomponents -Reliability, Pleasure, Stigma associated with condom use, embarrassment about negotiation and use of condom and about purchasing condom. Hundred truck drivers were recruited using convenient sampling and Mann-Whitney U and Kruskall Wallis Test were used to validate the subcomponents among those practicing unsafe sex.

**Results:**

Positive attitudes regarding reliability and effectiveness of condom were significantly higher among adult entrants and those resting more than 10 hours during journey whereas positive attitude regarding pleasure associated with condoms and stigma towards it is significantly higher among truck drivers travelled long distances and resting more than 10 hours.

**Conclusion:**

Adolescent entrants, those who have not travelled long distances and not rested more than 10 hrs requires improvement in the attitude towards condom use.

## Introduction

WHO reports globally 36.9 million people are living with HIV. 1In South-east Asia, 1.7 million people are living with HIV and number of people living with HIV in India is 2100000.^1^,^2^

Sexual promiscuous lifestyle is common among truck drivers and therefore susceptible of acquiring HIV infection. 3 Several worldwide studies have documented unsafe sexual practise among truck drivers.^4^–^9^ In India, the prevalence of HIV among truck drivers is 0.86%.^10^ Inconsistent condom use among truck drivers has been highlighted among Uganda truck drivers and Indian truck drivers.^11^,^12^

There seems to be few studies highlighting the attitude toward condom use among truck drivers. This study was undertaken with an objective to assess the attitude towards condom use and its predictors among truck drivers at transhipment location in Meerut district, India

## Materials and Method

### Study Design and Setting

This cross-sectional study was conducted at Trans-shipment area in Meerut district, India which is home to over two hundred transporters and over 500 trucks are available at any given point of time. Furthermore, a typical halt can range from thirty minutes to two days and work hours of most transporters are eleven hours.

### Sampling procedures and Sample size

Hundred truck drivers were recruited who were having a valid driver's license from the trans-shipment area using convenient sampling procedure.

### Study Tool and Procedure of Data collection

A pre-tested validated questionnaire comprising closed ended questions was used to document socio-demographic-occupational profile and for assessment of attitude towards condom use University of California Los Angeles Multidimensional Condom Attitude Scale (UCLA MCAS) was used.^13^

The truck drivers were briefed about the purpose of the study, importance of their participation, its consequential benefit to truck drivers and assurance of their confidentiality. After briefing them, the informed consent was taken from truck driver agreeing to participate and regardless of the participation, the truck drivers were given condoms and advices related to health issues. Truck drivers having wrong perception about condom were educated about the benefit of condom The pilot study, modification of questionnaire, recruitment of participants for main study and data analysis was done from a period of August 2014 to September 2015.

### Predictor Measures

#### Socio-demographic (Relationship status, education status)

**Occupation profile:** Type of truck drivers (based on distance traversed), average number of days away from home, average resting time during trip and type of entrant (adolescent or adult)

**Unsafe sex:** Our criterion for unsafe sex was operationally defined as inconsistent use of condom with high risky sexual partners like CSW and MSM irrespective type of intercourse-vaginal, anal or oral (Fellatio) and practicing either Cunnilingus or Analingus or both.

### Dependent measures

**Attitude towards Condom Use:** The 25 item UCLA MCAS 7-point Likert scale was used to assess the attitude towards condom use. The UCLA MCAS has 5 subscales (or domains) - Reliability and Effectiveness of Condoms, Pleasure associated with Condom, Stigma associated with condom use, Embarrassment about negotiation and use of condom and Embarrassment about purchasing condoms. 13

Reliability and effectiveness subscale included 1) Condoms are an effective method of preventing the spread of Aids and other sexually transmitted diseases 2) Condoms are unreliable (reverse scored) 3) I think condoms are an excellent means of contraception 4) Condoms do not offer reliable protection (reversed score) 5) Condoms are an effective method of birth control. Pleasure associated with condoms included 1) Use of condom is an interruption of foreplay (reversed score) 2) Condoms ruin the sexual act (reversed the score) 3) Condoms are a lot of fun 4) The use of condom can make sex more stimulating 5) Condoms are uncomfortable for both parties (reversed score)Stigma associated with Condom use included 1)Women think men who use condoms are jerk (reversed score) 2)If a couple is about to have sex and the man suggests using a condom, it is less likely that they will have sex (reversed score) 3) People who suggest condom use are a little bit geeky (reversed score) 4) Men who suggest using a condom are really boring (reversed score) 5) A woman who suggests using a condom does not trust her partner (reversed score). Embarrassment about negotiation and use of condom include 1) It is really hard to bring up the issue of using condoms to my partners (reversed score) 2) When I suggest using a condom I am almost always embarrassed (reversed score) 3) It is easy to suggest my partner that we use a condom 4) I never know what to say when my partner and I need to talk about condoms or other protection (reversed score) 5) I am comfortable talking about condoms with my partner. Embarrassment about purchasing condoms include 1) I always feel really uncomfortable when I buy condoms (reversed score) 2) I don't think that buying condoms is awkward 3) It is very embarrassing to buy condoms (reversed score) 4) It would be embarrassing to be seen buying condoms in a store (reversed score) 5) It would be embarrassing to be seen buying condoms in a store (reversed score)

### Data Analysis

The analysis was done in SPSS 21.

The total score for each item =

(Total response strongly agree * 7) + (Total response slightly agree*6) + (Total response can't say *5) + (Total response slightly agree*4) + (Total response slightly disagree* 3) + (Total response disagree*2) + (Total response strongly disagree*1) / Total Response (100)

Mean score% for each subscale = (mean score – 1)/(maximum value – minimum value)

Minimum value = 1 and maximum value = 7

The mean score for individual participant in different subscales was computed and non-parametric test [Kruskall Wallis and Mann-Whitney U Test] were applied to find the predictors of attitude regarding condom use.

### Ethical clearance

The study was a component of a dissertation and Subharti Medical College (Meerut, India) Institutional Committee approved the dissertation (SMC/PG-13/2013).

## Results

### Participant characteristics

Of the 100 participants, most were married (72%), literate (90%), adult entrant (69%), travelled long distance (77%) average time away from home less than 10 days (71%) and resting during journey less than 10 hours (65%).

### Attitude of Truck drivers for condom use

The attitude among truck driver regarding reliability and effectiveness of condom is 88.3% , pleasure associated with condom is 70.3% , stigma associated with condom is 73.7% , embarrassment about negotiation and condom use is 76% and purchasing condoms is 75.8%.

### Predictors of attitude of Truck drivers towards condom use

The mean score for attitude regarding reliability and effectiveness of condoms is significantly higher among adult entrants than adolescent entrants and those resting more than 10 hours during journey whereas mean score for regarding pleasure associated with condoms is significantly higher among truck drivers travelled long distances and resting more than 10 hours. Furthermore, the mean score for attitude regarding stigma associated with condom use is significantly higher among truck drivers travelled long distance and attitude regarding embarrassment about negotiation and use of condom was significantly higher among truck drivers resting more than 10 hours. However, mean score for attitude regarding embarrassment about purchasing condom use was significantly higher among adult entrants and those who have travelled long distance. [Table 1]

**Table 1:** Predictors of Attitude of Truck drivers towards condom use

	Reliability and effectiveness of condoms	P value	Pleasure associated with condoms	P value	Stigma associated with condom use	P value	Embarrassment about negotiation and use of condom	P value	Embarrassment about purchasing condoms	P value
**Age (N)**	Mean (standard deviation)		Mean (standard deviation)		Mean (Standard Deviation)		Mean (Standard deviation)		Mean (Standard deviation)	
= < 30(43)	6.36(.897)	0.467	5.30(1.23)	0.47	5.40(1.31)	0.393	5.572(1.40)	0.339	5.82(1.50)	0.324
31–40 (35)	6.20(.812)		5.29(1.49)		5.57(1.16)		5.76(1.12)		5.82(1.46)	
> 40 (22)	6.30(.806)		4.90(1.35)		5.19(1.16)		5.218(1.28)		5.44(1.37)	
**Relationship** **status**										
Married (72)	6.29(.88)	0.783	5.25(1.43)	0.413	5.45(1.28)	0.543	5.72(1.27)	0.06	5.88(1.49)	0.084
Unmarried (28)	6.31(.76)		5.12(1.13)		5.34(1.20)		5.14(1.33)		5.38(1.39)	
**Education status**										
illiterate (10)	6.52(.60)	0.592	5.96(1.21)	0.064	5.56(1.41)	0.501	5.08(1.55)	0.279	6.06(1.55)	0.275
Literate (90)	6.27(.86)		5.13(1.34)		5.40(1.21)		5.61(1.25)		5.71(1.418)	
Entrant Type										
Adolescent (31)	5.99(.98)	0.034	5.03(1.46)	0.403	5.135(1.29)	0.187	5.30(1.25)	0.156	5.25(1.63)	0.041
Adult (69)	6.43(.74)		5.30(1.29)		5.547(1.18)		5.67(1.29)		5.96(1.27)	
**Distance** **Travelled**										
Not Travelled Long distances (23)	6.03(.925)	0.092	4.56(1.17)	0.005	4.99(1.29)	0.049	5.25(1.42)	0.197	5.17(1.47)	0.024
Travelled Long distances (77)	6.37(.81)		5.41(1.34)		5.54(1.18)		5.65(1.23)		5.91(1.37)	
**Time away from** **home**										
Average time away from home less than 10 days (71)	6.31(.83)	0.961	5.19(1.41)	0.84	5.52(1.21)	0.271	5.63(1.26)	0.382	5.83(1.39)	0.293
Average time away from home more than 10 days (29)	6.26(.89)		5.27(1.20)		5.17(1.24)		5.37(1.35)		5.52(1.50)	
**Resting time** **during journey**										
Resting time less than 10 hours (65)	6.16(.89)	0.033	5.0092(1.26)	0.018	5.27(1.28)	0.105	5.36(1.27)	0.027	5.55(1.48)	0.085
Resting time more than 10 hours (35)	6.55(.689)		5.60(1.44)		5.68(1.06)		5.93(1.24)		6.09(1.25)	
**Unsafe sex** **Practice**										
No (62)	6.3(.88)	0.653	5.23(1.31)	0.912	5.31(1.27)	0.219	5.56(1.31)	0.92	5.68(1.52)	0.851
Yes (38)	6.28(.78)		5.18(1.42)		5.60(1.12)		5.55(1.25)		5.84(1.26)	

## Discussion

This seems to be the first study for elaborative assessment of attitude toward condom use among truck driver population in India using UCLA Multidimensional Condom Attitude Scale. The findings of the study indicate in almost all domains the attitude of truck driver was far from satisfactory.

This scale was used among 20 recruited community members assessing difference in attitude towards condom use between those consuming alcohol and does not consuming it.^14^ The pleasure subscale of MCAS was used to assess the attitude in a study with an objective to determine rates of sex- and drug-related risk-taking in a sample of HIV-positive injection 50 HIV-seropositive injection drug users entering methadone maintenance treatment. This study revealed among sexual risk takers scored significantly higher on the perception that condom reduces pleasure.^15^ Its use has been documented to identify attitude correlates toward condom use among 270 drug users which revealed positive attitude regarding condom effects on pleasure and not feeling embarrassed about negotiating condom use was associated with higher likelihood to use condom.^16^ Moreover, it was used in studying the correlates of Unprotected Sex Among Adult Heterosexual Men Living with HIV , where findings indicate unprotected sex was inversely associated with positive attitude towards condom use.^17^ Furthermore, its use has been documented in a study assessing influence of partner participation on sexual risk behaviour reduction among HIV positive Zambian women where the findings indicate women with greater partner involvement had higher rates of condom use and more positive attitudes regarding condom use.^18^

The study is subjected to limitation because of small sample size recruited using convenience sample and social desirability bias may have influence on the response of the participants. Hence it is recommended the study be conducted on a large sample size using probability sampling.

## Conclusion

The age of the entrant, travelling long distances and resting time more than 10 hours were predictors significantly affecting attitude of truck drivers regarding condom use.
